# The Role of Psychedelics in the Treatment of Substance Use Disorders: An Overview of Systematic Reviews

**DOI:** 10.3390/brainsci15101056

**Published:** 2025-09-28

**Authors:** Sabrina Correa da Costa, Nicholas L. Bormann, Tyler Oesterle, Michele T. McGinnis, Ming-Fen Ho, Sara A. Vettleson-Trutza, Teresa Rummans, Mark S. Gold

**Affiliations:** 1 Department of Psychiatry and Psychology, Mayo Clinic, Rochester, MN 55905, USA; bormann.nicholas@mayo.edu (N.L.B.); oesterle.tyler@mayo.edu (T.O.); ho.mingfen@mayo.edu (M.-F.H.); 2 Mayo Clinic Libraries, Mayo Clinic, Rochester, MN 55905, USA; mcginnis.michele@mayo.edu; 3 Department of Neurologic Surgery, Mayo Clinic, Rochester, MN 55905, USA; vettleson-trutza.sara@mayo.edu; 4 Department of Psychiatry and Psychology, Mayo Clinic, Jacksonville, FL 32224, USA; rummans.teresa@mayo.edu; 5 Department of Psychiatry, Washington University in St. Louis, St. Louis, MO 63130, USA; drmarksgold@gmail.com

**Keywords:** substance use disorder (SUD), addiction, ketamine, psychedelics, hallucinogens, treatment, interventional psychiatry

## Abstract

**Background**: Substance use disorders (SUDs) are highly prevalent, affecting over 48.5 million Americans. Available treatments for SUD remain insufficient, and many patients do not respond to existing interventions despite adequate adherence to treatments. While novel therapies for SUD are urgently needed, the use of psychedelic drugs for the treatment of SUDs has shown promise. **Objectives**: This overview of systematic reviews summarizes existing evidence on hallucinogens—serotonergic psychedelics and ketamine—for the treatment of SUD. **Methods:** A comprehensive search of the literature was conducted to identify relevant evidence for using serotonergic and non-serotonergic psychedelics for the treatment of SUDs. After initial screening (*n* = 468 studies), 62 studies were retrieved and assessed for eligibility, and a total of 16 systematic reviews were included. **Conclusions:** Although preliminary, evidence suggests that the use of serotonergic and non-serotonergic psychedelics for the treatment of SUD may provide advantages over traditional therapeutics, and these compounds may eventually become part of the next generation of treatments for SUD under specific circumstances. Research with these drugs has faced significant challenges, though, and caution when interpreting results is warranted, given high risk of bias and several other methodological limitations from the studies to date. Furthermore, risks associated with these drugs are not negligible. For now, the use of psychedelic drugs for the treatment of SUDs remains experimental, and existing evidence is insufficient to support its use in clinical practice.

## 1. Introduction

Substance use disorders (SUDs) continue to pose a major public health challenge, contributing to substantial morbidity, disability, and premature mortality in the US and worldwide [1,2,3,4]. SUDs are highly prevalent, affecting over 48.5 million individuals, 17.1% of the US population, according to the National Survey of Drug Use and Health (NSDUH) [2,5]. Importantly, alcohol and opioids remain leading causes of preventable death, disproportionately affecting young individuals and, altogether, claiming nearly 300,000 lives in the US in 2021 alone [6]. Problematic alcohol use is implicated in 18.5% of emergency department visits each year, 20% of prescription opioid overdose fatalities, whereas 21% of suicide decedents have blood alcohol concentrations of 0.1% or higher [7,8,9]. SUDs are also associated with significant personal and societal burdens greatly attributed to health-related costs, disability and loss of productivity, and valuation of loss of quality of life and life lost due to fatal drug overdoses [1,10,11].

While existing treatments have helped many individuals experiencing SUDs, available interventions remain limited to a few Food and Drug Administration (FDA)-approved medications, often used as stand-alone or in combination with other interventions. To date, no approved pharmacotherapies exist outside of alcohol, nicotine, and opioid use disorder treatments, adding complexity to already challenging clinical scenarios [12]. Furthermore, available treatments for SUD remain insufficient, and many individuals only experience modest benefits despite adequate adherence to treatment [12]. Consequently, many persons suffering from SUD and other addictions remain highly symptomatic, untreated or undertreated. Relapse rates in SUD are unsurprisingly high, and a sizable number of affected individuals will eventually experience illness progression, treatment refractoriness, neuropsychiatric and other medical complications, non-fatal overdoses, and, ultimately, disability or even death [5,13,14].

In recent years, interventional and procedural approaches in psychiatry have expanded. Neuromodulatory treatments, such as repetitive transcranial magnetic stimulation (rTMS), transcranial direct current stimulation (tDCS), and deep brain stimulation (DBS) have become more widely applied to psychiatric conditions, whereas ketamine treatment has been more extensively prescribed [15,16,17]. While rTMS FDA-approval for smoking cessation represents one of the most significant advances in SUD treatment in recent years, novel therapies for SUD and other addictions are urgently needed, and the use of hallucinogens, particularly classic psychedelics and ketamine, has shown promise [18,19]. It is important to note that the literature on psychedelic drugs for SUDs, although expanding, remains diffuse, spread across multiple reviews with varying methodological rigor, inconsistent interventions, and substance-specific focus, making it difficult to appraise where evidence converges and how best to inform practice and future research [20,21]. Thus, this overview of systematic reviews aims to consolidate existing syntheses on the benefits of hallucinogens—serotonergic or classical psychedelics, ketamine, and (3,4-Methylenedioxymethamphetamine) MDMA—for the treatment of SUDs. The goal is to highlight the potential benefits of these drugs along with gaps and key limitations across studies, identifying priority areas for future investigation, clinical and translational research.

## 2. Materials and Methods

### 2.1. Psychedelic Classification

From a pharmacological perspective, hallucinogens are classified into the following: serotonergic or classical psychedelics, which exert their primary effects via agonism at serotonin 5-HT_2A_ receptors (e.g., lysergic acid diethylamide [LSD], psilocybin, and N,N-dimethyltryptamine [DMT]), non-serotonergic compounds, such as ketamine and phencyclidine (PCP), which act primarily as N-methyl-D-aspartate (NMDA) receptor antagonists, and atypical agents, such as MDMA, a synthetic empathogen with a complex pharmacology and both stimulant and hallucinogenic effects further classified as phenylethylamine [21,22,23,24,25] (Table 1).

While the precise neurobiological mechanisms of these compounds remain incompletely understood, accumulating evidence suggests complex psychopharmacology and distinctive effects on perception, cognition, and consciousness [21,22,23,24,25]. Substances such as mescaline, ayahuasca, and psilocybin are naturally occurring examples of psychedelics, some of which have been used for millennia in religious and spiritual ceremonies [26,27]. while ketamine has been used in human and veterinarian medicine as a dissociative anesthetic for several decades [28,29,30,31,32].

Besides serotonergic effects, psychedelic drugs seem to engage dopamine D2 receptors, kappa opioid receptors, and monoamine oxidase enzymes, and influence monoamine transmission via modulation of serotonergic, dopaminergic, and noradrenergic transporters [24,33]. These broad and complex mechanisms are believed to contribute to both their acute subjective effects besides their more pervasive modulation of neural circuits implicated in mood, cognition, and reward, pharmacological effects particularly relevant in substance use and addictions.

Ketamine, although not a classical serotonergic hallucinogen, shares the clinical and pharmacological features of psychedelics, thus classified as a hallucinogen drug [34]. Ketamine acts primarily as a non-competitive NMDA receptor antagonist; however, it also modulates glutamatergic neurotransmission and influences dopaminergic and serotonergic circuits through effects on GABAergic interneurons [5,6,7]. Clinically, this manifests as a dissociative anesthetic, characterized by preserved respiratory drive, hemodynamic stability, and altered states of consciousness [8,9,10]. Interestingly, recent evidence suggests that ketamine may also exert its pharmacological effects though modulation of opioid pathways and signaling, as evidenced by attenuation of its rapid antidepressant effects by opioid receptor antagonism, which seems to be independent of its dissociative effects [35,36]. Famula et al. critically examined the neurobiological underpinnings of ketamine’s potential therapeutic effects in SUD, highlighting its impact on glutamatergic neurotransmission, neuroplasticity, and reward pathways, providing insights into potential mechanisms that may contribute to its effectiveness in treating SUDs [37].

While psychedelic drugs are currently classified as class I substances, denoting no current accepted medical use and high potential for abuse, ketamine falls into a class III drug classification by the US Drug Enforcement Agency (DEA), denoting moderate to low potential for abuse, while its medical use is recognized [38,39]. Throughout the years, ketamine’s clinical applications have expanded into chronic pain management, rapid-acting antidepressant and anti-suicidal effects, post-traumatic stress disorder (PTSD), bipolar disorder, obsessive–compulsive disorder (OCD), and anxiety disorders [21]. Emerging evidence also suggests ketamine’s efficacy in managing SUDs, such as alcohol, cannabis, cocaine, and opioid use disorders [40,41,42,43].

### 2.2. Search Strategy and Methods

#### Overview of Systematic Reviews

A comprehensive search of the literature was conducted to identify relevant evidence on hallucinogens—psychedelics and ketamine—for the treatment of SUDs (please refer to “Supplementary Materials” for a detailed search strategy report).

This study is reported in accordance with the PRISMA-S, an extension to the PRISMA statement for reporting literature searches in systematic reviews [44]. A medical librarian (MM) deployed the search strategy in consultation with the research team. Studies were identified from searches of the databases Embase, MEDLINE, Cochrane Central Register of Controlled Trials (CENTRAL), and the Cochrane Database of Systematic Reviews (CDSR), and PsycInfo—all via the Wolters Kluwer Ovid interface; Scopus; Science Citation Index Expanded (SCI-Expanded) and Emerging Sources Citation Index (ESCI)—both via the Clarivate Analytics Web of Science interface. The Embase search was manually translated across all resources using syntax, controlled vocabulary, and search fields. MeSH thesaurus terms from MEDLINE, EMTREE thesaurus terms from Embase, Thesaurus of Psychological Index Terms, and free and text words were used for search concepts.

Inclusion criteria: The literature time range encompassed studies from 1946 to 2024. English-language database limits were applied as available or built into searches whenever possible. An unpublished filter was used to limit the study design to systematic reviews with or without meta-analysis on interventions of interest—serotonergic and non-serotonergic drugs for the treatment of SUDs. Conference abstracts and conference reviews were excluded.

Titles and abstracts were initially screened for relevance by two independent reviewers, then full-text reviews were conducted for final inclusion. Disagreements were resolved by discussion, with a third-party tiebreaker activated if needed. All records were downloaded or manually added to EndNote 20 Desktop version.

## 3. Results

**Figure 1:**
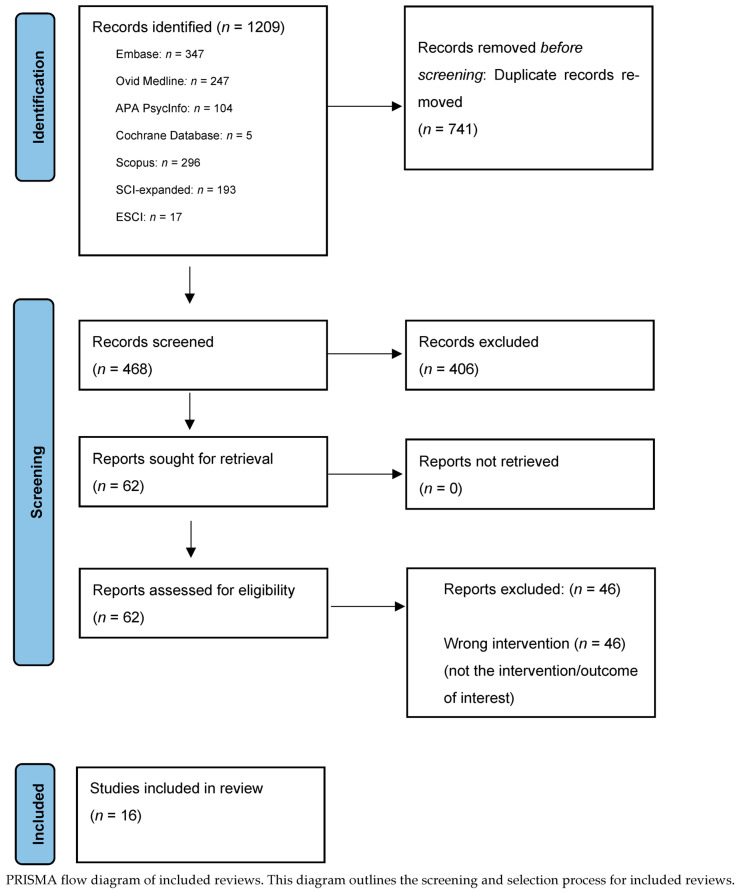
PRISMA flow diagram.

Initial database searches (Figure 1*—PRISMA flow diagram*) were performed on *n* = 1209 studies; 741 duplicates were identified and removed, resulting in 468 studies for initial screening, out of which 406 studies were excluded. Sixty-two studies were retrieved and assessed for eligibility, and a total of 16 systematic reviews that examined the therapeutic use of hallucinogens for SUD treatment were identified: 10 reviews focused on serotonergic psychedelics, including LSD, psilocybin, MDMA, ayahuasca, mescaline, and ibogaine (Table 2) [45,46,47,48,49,50,51,52,53,54], while 6 systematic reviews specifically investigated the use of ketamine for the treatment of SUDs (Table 3) [41,55,56,57,58,59].

### 3.1. Psychedelic Drugs—Psilocybin, LSD, Ayahuasca, Ibogaine, MDMA, Mescaline

Across the 10 included reviews [45,46,47,48,49,50,51,52,53,54], psychedelics were evaluated for their effects on various substance use outcomes such as abstinence, relapse rates, cravings, withdrawal symptoms, and other substance-related behaviors. Preliminary evidence suggests that psychedelic drugs may hold promise in the treatment of SUD with varying levels of efficacy across substances and conditions. Importantly, the strength and consistency of evidence varied by SUD and compound (Table 2).

Interestingly, the therapeutic benefits of psychedelic drugs were frequently associated with or attributed to the “psychedelic” or “mind manifesting” and “mystical” experiences, with participants reporting shifts in self-awareness, emotional processing, and well-being. Notably, in the 1950s, Alcoholic Anonymous (AA) founder Bill Wilson experimented with LSD in a controlled, medically supervised setting after being introduced to it by British psychiatrist Dr. Humphry Osmond, a pioneer in psychedelic therapy [60]. Wilson believed LSD could help “alcoholics” achieve the “spiritual awakening” necessary for recovery. He felt that LSD temporarily removed the ego barrier and allowed a sense of connectedness, which mirrored the “spiritual experience” described in AA’s 12th Step. Unsurprisingly, the majority of AA’s leadership and membership at the time were opposed to Wilson’s interest in LSD, viewing it as inconsistent with AA’s principle of sobriety [61,62,63].

For **alcohol use disorder** (AUD), LSD demonstrated greater reductions in alcohol-induced subjective effects compared to psilocybin, with one study finding that 83% of participants no longer met the criteria for AUD following an LSD-induced psychedelic experience [50]. Mescaline showed a contrasting pattern, being linked to increased alcohol consumption in youth (adolescents and college students), while 76% of adults with past AUD reported improvements in alcohol use outcomes following mescaline use in naturalistic settings [45]. Psilocybin for AUD has shown mixed results. Calleja-Conde et al. examined the relationship between classic psychedelics and alcohol consumption. They identified 27 studies, 20 of which were human studies, suggesting that classic psychedelics could help reduce alcohol consumption. The authors identified methodological concerns, such as a small number of participants, lack of control groups, and difficulty in isolating the effect of classic psychedelics [45]. Similarly, Sicignano et al. found that at the first recorded follow-up, LSD [*n* = 3, Odds Ratio (OR) 1.99 (95% Confidence interval (CI): 1.10 to 3.61)] and any psychedelic drugs [*n* = 4, OR 2.16 (95%CI: 1.26 to 3.69)] enhanced the odds of participants achieving abstinence or a substantial reduction in alcohol consumption versus placebo in randomized, double-blind, placebo-controlled trials [46]. When the inclusion criteria were relaxed to include controlled trials without double-blinding or placebo control, LSD [*n* = 5, OR 1.79 (95%CI: 1.36 to 2.34)] and any psychedelic therapy [*n* = 6, OR 1.89 (95%CI: 1.42 to 2.50)] still enhanced the odds of patients achieving abstinence or a substantial reduction in drinking alcohol [46]. However, 4 of 6 trials had high risk of bias and other methodological issues [46].

In a mixed methods systematic review, Sharma et al. reviewed evidence from 10 papers and 7 studies investigating the use of psilocybin, ibogaine, and ayahuasca, alone or adjunctive to psychotherapy for a wide range of addictions [53]. Measures of abstinence, substance use, psychological and psychosocial outcomes, craving, and withdrawal reported promising results [53]. However, the authors underscored insufficient evidence to suggest the effectiveness of any of the psychedelics on any specific SUD or misuse besides methodological limitations across studies [53].

Van der Meer et al. identified one RCT and three small clinical trials assessing the efficacy of psilocybin combined with some form of psychotherapy for alcohol and tobacco use disorder. While all four clinical trials indicated the positive effects of psilocybin-assisted therapy, authors concluded that larger studies are needed. Notably, the percentage of heavy drinking days during the 32-week double-blind period was significantly lower for psilocybin compared to placebo (mean difference of 13.9, 95% CI = 3.0–24.7, *p* = 0.01) in a RCT included. For **tobacco use disorder**, in a pilot study (*n* = 15) included, the 7-day point prevalence of smoking abstinence at 26 weeks was 80% (12/15), and at 52 weeks 67% (10/15) [52]. Rodrigues et al., examining the effects of ayahuasca and its alkaloids on SUD, found that ayahuasca was associated with lower alcohol consumption, reduced prevalence of AUD and tobacco and significant decrease in dependency [49]. Lastly, Mosca, A. et al. examined the efficacy of ibogaine/noribogaine for the treatment of SUDs and found short-term benefits, including improved mood, reduced cravings, and alcohol and other drug use cessation; however, cardiotoxicity (QTc prolongation, Torsade de Pointes, arrhythmias) and mortality were of significant concern [47].

The evidence on the efficacy of psychedelics for other SUDs seems sparse. For **opioid use disorder** (OUD), ibogaine has demonstrated mixed results, with research participants reporting reduced withdrawal symptoms and sustained remission from opioid use for extended periods of time [47]. Phan, A. N. and G. E. Terry systematically reviewed the effects of ketamine, MDMA, lorcaserin, and LSD on **cannabis use and use disorder** (CUD) and found no significant impact on cannabis use with MDMA, while LSD seemed to be associated with reduced cannabis use [51]. Spoelstra, S. K. et al. found that the majority of studies on psychedelics for the treatment of **tobacco use disorder** focused on psilocybin, although studies have also been conducted on ayahuasca, mescaline, peyote, LSD, lysergic acid amide (LSA) and dimethyltryptamine (DMT) for smoking cessation [48]. The authors concluded that there is evidence that psychedelics, in particular psilocybin, may offer a potential avenue for the treatment of tobacco use disorder, but the evidence for these psychedelics is too small to draw definitive conclusions [48].

### 3.2. Ketamine

Kelson et al. conducted a systematic review of 11 studies with 854 patients total evaluating ketamine as a treatment for **AUD** [55]. Combination therapy with ketamine and psychotherapy yielded the highest abstinence rates, with up to 70% of patients maintaining abstinence. While ketamine’s impact on withdrawal symptoms was mixed, there is evidence suggesting a favorable impact on benzodiazepine dose requirement, ICU (5.7 versus 11.2 days), decreased likelihood of intubation and shorter length of hospital stay [12]. Most patients also reported significant reductions in alcohol cravings, though results varied based on treatment protocol [55]. Similarly, Garel et al. analyzed 634 patients with AUD across eight studies and found that ketamine, either monotherapy or combined with psychotherapy, reduced both short-term (10 days) and long-term (up to 3 years) outcomes of alcohol consumption, namely abstinence, time to relapse, heavy/binge drinking [57]. However, the effects on cravings were inconsistent.

Jones et al. reviewed seven studies on ketamine’s role across multiple SUDs, including AUD, and found that ketamine was associated with abstinence, in some cases up to two years post a single ketamine infusion [41]. Moreover, ketamine was linked to lower relapse rates, enhanced motivation for sobriety, and reduced compulsive alcohol-seeking behaviors. In this study, the authors hypothesized that ketamine’s glutamatergic modulation and neuroplasticity effects may help brain connectivity and “rewire” maladaptive drinking patterns. While some studies suggested that ketamine alleviates severe withdrawal symptoms like *delirium tremens*, its impact on general withdrawal symptoms remains unclear. Moreover, in this review, Jones et al. also examined the effects of ketamine on opioid, cocaine, and cannabis use disorders [41]. Two clinical studies on **cocaine use disorder** (total of *n* = 28 participants; first study: *n* = 8 non-treatment and non-abstinence seeking adults; second study: *n* = 20 non-treatment seeking) found that ketamine treatment helped individuals reduce cocaine consumption, with effects observed as early as the same day and lasting up to four weeks at follow-up [41]. Additionally, patients with cocaine use disorder reported increased motivation to abstain from drug use. Notably, small sample sizes and other methodological challenges limit the generalizability of these findings [41]. Furthermore, ketamine for **OUD** has shown promising outcomes, with higher ketamine doses seeming to be more effective. However, various included studies lacked control groups and did not integrate medication for opioid use disorders (MOUD) and standardized treatments, such as buprenorphine or methadone, for comparison making it difficult to assess ketamine’s relative effectiveness to well established treatments for OUD [41,59]. Following these encouraging but limited findings, clinical trials investigating ketamine use for **cannabis use disorder** and **cannabis withdrawal symptoms** have been initiated, although its efficacy is yet to be determined [41].

## 4. Discussion

Also known as “*umbrella review*,” “*review of reviews*,” and “*metareview*,” an overview of systematic *reviews* is a relatively new method of evidence synthesis that collates and organizes information from multiple systematic reviews with the goal to summarize, compare and contrast evidence that can guide clinical and research endeavors, identifying gaps in the literature and areas for future research [64]. It is a systematic approach that can be particularly useful when there is significant heterogeneity in the literature.

Through this novel approach, this overview of systematic reviews consolidates existing syntheses on psychedelic drugs—serotonergic psychedelics and ketamine—for the treatment of SUDs. The goal was to highlight the potential benefits of these drugs along with key limitations across studies, identifying priority areas for future investigation, clinical and translational research. Overall, the reviews presented here underscore the therapeutic potential of both serotonergic and non-serotonergic psychedelics particularly for alcohol use disorder, while evidence is much sparser for other SUDs.

The mechanisms of action underlying the therapeutic benefits of hallucinogens for SUD and other mental health conditions remain poorly understood, in part due to its heterogeneity and complex psychopharmacology. While preclinical studies have provided significant insights into mechanisms of action, isolating biological markers and translating these findings into more refined drug development and clinical application remain a challenge.

While advantages of psychedelic drugs and ketamine over treatment as usual may include single-dose interventions over complex treatment regimens, rapid onset of action and more sustained clinical benefits, evidence to date remains equivocal due to multiple methodological limitations, such as small sample sizes, study designs, risk of bias, including masking interventions, and lack of comparisons with established treatments and standards of care. However, if preliminary findings are consistently replicated by methodologically sound studies, these drugs may eventually represent alternatives to traditional medications or be used adjunctive to existing pharmacological and non-pharmacological treatments for optimal outcomes and enhanced treatment response.

Importantly, it is also possible to consider these interventions as alternatives or adjunctive strategies in more severe and refractory conditions, particularly in the setting of co-occurring disorders. For instance, while contemporary research with MDMA has shown significant reduction in PTSD symptoms, granting MDMA FDA “breakthrough therapy” designation for PTSD in 2017, little is known about the effects of MDMA-AT on alcohol consumption in this population. Considering the high co-occurrence of PTSD-AUD and preliminary evidence on the benefits of psychedelics also for SUD, secondary analysis from a phase 3 trial has demonstrated that, compared to placebo + therapy, MDMA-AT was associated with a significantly greater reduction in mean AUDIT score changes [65]. Thus, future research on the effects of MDMA-AT as an integrated treatment for co-occurring PTSD-AUD is warranted. Similarly, it remains unclear whether alcohol and other substance use outcomes would vary for individuals with co-occurring treatment-resistant depression and SUD receiving ketamine therapy, and future research should explore these questions. Unfortunately, to date, most clinical trials, whether on interventions for SUD or other psychiatric conditions, almost invariably exclude comorbid conditions, limiting our understanding on the impact of interventions for individuals suffering from co-occurring disorders.

While promising, much is yet to be performed. As aforementioned, significant **methodological limitations** remain an area of great concern, limiting the interpretation of results and generalizability of findings. Given the unique and profound psychoactive effects of these drugs, masking interventions remains a challenge. Heterogeneity of treatment protocols and psychosocial interventions also limit outcomes and appraisal of results. Most trials have incorporated adjunctive behavioral/psychotherapeutic interventions, making it difficult to isolate the “hallucinogenic effects.” The absence of reliable active placebos further complicates the interpretation of findings, as both research participants and investigators are often able to accurately predict treatment allocation. Thus, high risk of bias from included studies besides low-quality level of evidence to support hallucinogen therapies for SUD should not be ignored. Finally, ensuring fair subject selection and equitable study participation are critical for ethical human research and generalizability of findings.

**Safety concerns**, including autonomic instability, cardiovascular and respiratory side effects, anxiety, agitation, dysphoria, confusion, perceptual disturbances and dissociative symptoms have been extensively reported, although claimed to be dose-dependent and not life-threatening, somewhat contrary to findings from studies on ibogaine and noribogaine [47]. Challenges related to real life situations, such as elucidating drug–drug interactions, risks of serotonin syndrome, neurodevelopmental consequences, and the use of ketamine and psychedelic-assisted therapy in special populations, such as individuals with psychotic disorders, polypharmacy, older adults and geriatric populations, and safety in individuals with SUD, are yet to be clarified. Long-term effects and potential negative consequences are too to be determined. Misuse potential, particularly of ketamine, has been extensively reported since ketamine has been used for medical purposes, initially constrained to healthcare professionals and individuals with access to this drug, although non-medical ketamine has reached the streets over the decades, now. Medical complications from non-medical ketamine use and chronic exposure, such as cardiovascular disease, uropathy (ulcerative cystitis, hydronephrosis, chronic kidney disease and renal failure), neurocognitive impairment, unintentional accidents and injuries, and deaths have been reported.

Notably, non-medical use of ketamine has dramatically increased in recent years, particularly in Eastern Europe, The United Kingdom, Australia, South and East Asia, but also in the US. Ketamine-related ER visits and post-mortem toxicology reports are an area of concern. Deaths attributed to ketamine and other hallucinogens have been reported, including high profile cases in media outlets. The co-ingestion of ketamine and other drugs (e.g., MDMA, cocaine, and gamma-hydroxybutyrate—GHB) is also common, particularly in specific settings (e.g., night clubs and raves) and among specific populations, which poses even higher risks of toxicity and multiple medical complications than ketamine alone. Available formulations—powder, liquid, or capsules, most commonly used by nasal insufflation—are relatively inexpensive and have become more easily accessible in recent years under the street names of “Cat Tranquilizer, Cat Valium, Jet K, Kit Kat, Purple, Special K, Special La Coke, Super Acid, Super K, Vitamin K,” according to the DEA [56,66,67,68,69,70,71,72,73,74,75,76,77,78,79].

Thus, while the benefits of hallucinogens for psychiatric conditions, including SUD treatment, are promising, risks and potential harm should not be ignored. The medical use of scheduled drugs, such as cannabis, psychedelics, and ketamine has historically lowered the public’s perception of harm from these drugs, leading to substantial increase in its consumption in unsupervised medical settings and for non-medical purposes. As importantly, predatorial and careless ketamine clinics, retreats, and “medical” practices have proliferated in recent years. Finally, the devaluation or demonization of FDA-approved psychotropic medications has caused a great disservice to the larger public.

To our knowledge, this overview of systematic reviews is the first study to integrate and synthesize evidence from both serotonergic (psychedelics) and non-serotonergic (ketamine) hallucinogens for SUD treatment. It highlights results from multiple studies on potential clinical benefits of hallucinogens for SUDs and underscores gaps in the existing literature and areas for future research implementation, such as study design and methodology. Similarly to other reviews on these topics, the heterogeneity of studies, small sample sizes, and high risk of bias still represent limitations not only of this study but also the evidence to date. It is also important to note that the effects of psychotherapeutic interventions for SUD have been understudied, particularly in the setting of psychedelic- and ketamine-assisted psychotherapy. The disentangling effects of pharmacological from behavioral interventions is complex yet warranted. Hopefully, the results presented here will be further investigated by larger and methodologically sound clinical trials, involving broader and more representative patient populations, besides assessing real-life situations before these drugs are considered ready for prime time.

## 5. Conclusions

As the pursuit of novel and more effective treatments for SUD continues, findings from pre-clinical and human studies with hallucinogens have shown promise. The scientific value of psychedelics and ketamine as novel strategies for the treatment of addictions seems compelling, and the advantages of these drugs over treatment as usual may include single-dose interventions over complex pharmacological regimens, likely enhancing efficacy and adherence. Nonetheless, research with these compounds has faced significant challenges, and the many risks associated with these drugs are not negligible. Great caution when interpreting results is warranted, given the high risk of bias and several methodological limitations from studies to date. These limitations highlight the need for more rigorous clinical trials to further elucidate treatment response, efficacy and the long-term safety of hallucinogens, particularly for individuals with SUDs. Unarguably, the results of psychedelic-assisted therapy, particularly for the treatment of alcohol use disorder and smoking cessation, have shown remarkable short- and long-term effects, some unparallelled with traditional pharmacological interventions. While several trials are underway, if preliminary results are reliably replicated, psychedelic drugs and ketamine may become part of the next generation of treatments for SUDs. For now, the use of psychedelic drugs and ketamine for the treatment of SUDs remains experimental, and existing evidence is insufficient to support its use in clinical practice.

## Figures and Tables

**Table 1:** Basic pharmacology and classification of hallucinogens.

Class	Molecular Structure	Pharmacology and Background
***Ergolines*** LSD * semisynthetic	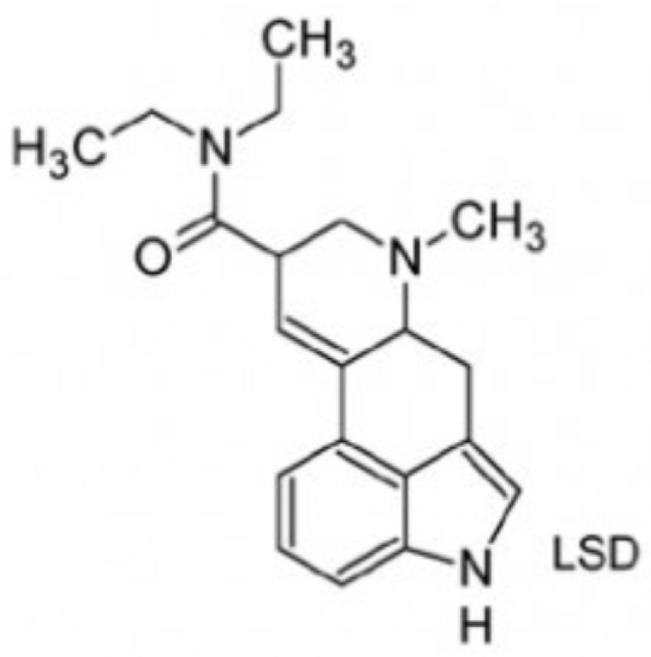	Semisynthetic, derived from the class of ergolines (*Claviceps purpurea*). Mechanism of action—primarily mediated by the serotonergic agonism (5HT-1A and 5HT-2A) in the dorsal raphe and effects on dopaminergic, Trace Amine Associate receptor 1 (TAAR_1_) and 5HT-2A systems in the ventral tegmental area. LSD has high potency and high affinity for 5HT-2A receptors. First synthesized by the Swiss scientist Albert Hofmann in 1938, LSD was marketed under the brand name of Delysid^®^ (LSD 25) until 1965, when Sandoz removed it from the market due to extensive non-medical use and experimentation with this drug.
***Indole ethylamines*** **psilocybin** (“magic mushrooms”) **DMT** (Psychotria viridis, Mimosa hostilis) natural—plant-based hallucinogens	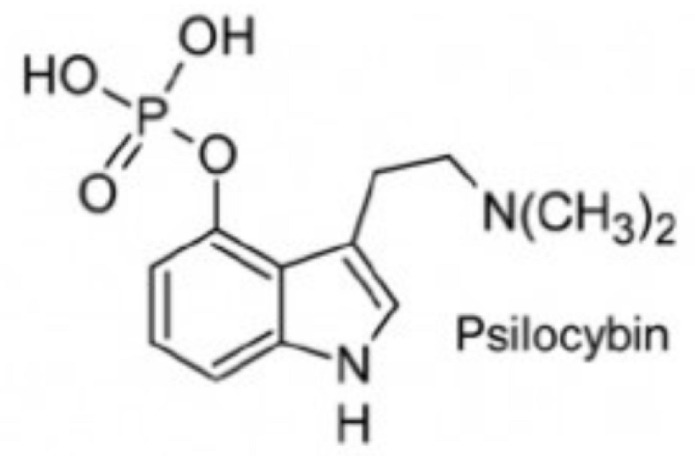 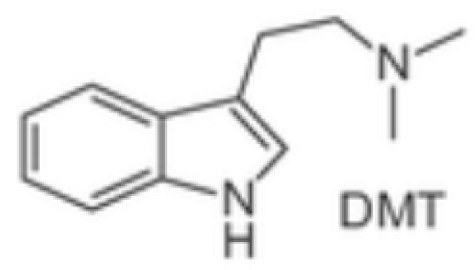 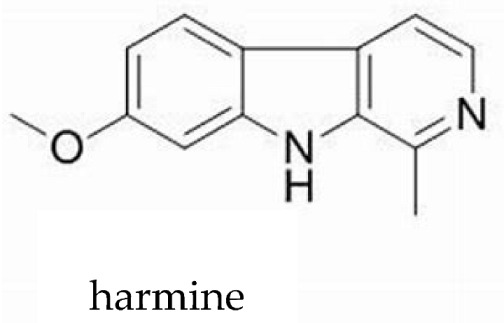	Psilocybin—naturally occurring alkaloid from the class of indole ethylamine present in over 200 different species of fungus also known as “magic mushrooms.” Psilocybin has been used as an entheogenic ** substance in religious and sacramental ceremonies by South and Central American natives for centuries. Curiously, the Swiss scientist Albert Hofmann, who synthesized LSD in 1938, also isolated psilocybin. Similarly to LSD, psilocybin was also marketed by Sandoz in the 1950s under the brand name Indocybin^®^. Ayahuasca is a decoction prepared from *Banisteriopsis caapi* and a dimethyltryptamine (DMT), used by Indigenous cultures in South America (Brazil, Peru, Colombia and Ecuador) as part of traditional medicine and religion (Santo Daime, Uniao do Vegetal). Ayahuasca’s psychoactive effects derive mainly from DMT, which is only made possible through the combination of *Banisteriopsis caapi* and its effects as a selective and reversible MAO-A inhibitor, preventing DMT degradation by MAO-A.
***Phenylethylamines*** **Mescaline,** natural—plant-based **MDMA**, synthetic *	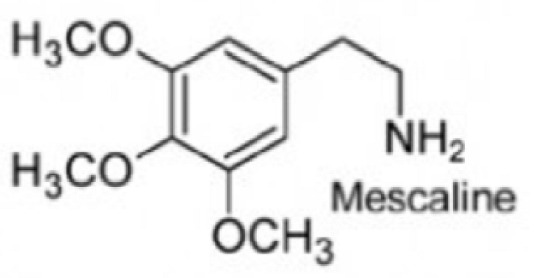 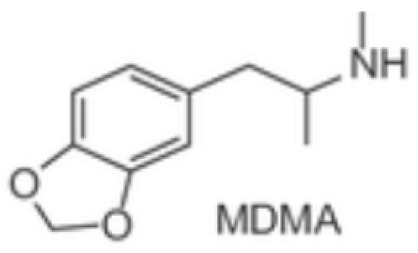	Naturally occurring, mescaline is a psychedelic alkaloid found in cacti like peyote used for medical, recreational, and spiritual purposes in the US, Mexico, and South America. First scientific research with psychedelics is believed to date back to 1897, when Arthur Heffter initially isolated mescaline from the peyote cactus and further characterized its psychoactive properties. MDMA is a synthetic empathogen or entactogen drug with a complex pharmacology and both stimulant and hallucinogenic effects. First synthesized in 1912 by chemist Anton Kollisch, MDMA likely holds the highest potential for misuse, being scheduled as class I by the DEA in 1985. MDMA is also known as “ecstasy” or “molly” and considered a “club drug” commonly used in raves, festivals, and clubs.
***Arylcyclohexylamine*** **Ketamine** synthetic	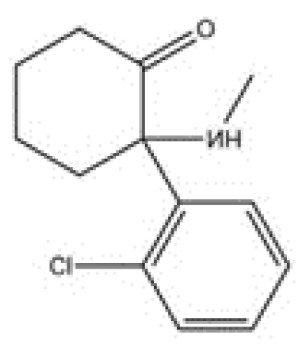	Synthetic noncompetitive NMDA-r antagonist; ketamine also modulates glutamatergic neurotransmission and influences dopaminergic and serotonergic circuits through effects on GABAergic interneurons. It seems to exert effects on mu, kappa, and delta opioid receptors. Ketamine is a dissociative anesthetic synthesized in the early 1960s by Parke-Davis as a replacement for phencyclidine. Ketamine has been extensively used in human and veterinarian medicine

LSD—lysergic acid diethylamide; DMT—N, N-dimethyltryptamine; MDMA—3,4-methylenedioxy-methylamphetamine. MAO—monoamine oxidase. * Empathogens or entactogens (“en”—Greek—“within,” “tactus”—Latin—“touch,” “gen”—Greek—“generate”)—are believed to have the potential to enhance closeness and connectedness, ability to decrease anxiety, increase trust and self-acceptance. ** Entheogen—God within (En—Greek—“within,” Theos—Greek—“God,” gen—Greek—“produce”)—used in spiritual or religious rituals.

**Table 2:** Psychedelics and serotonergic hallucinogens.

Authors	Target Condition	Inclusion/Exclusion Criteria	Intervention	Outcomes
**Javier Calleja-Conde et al. [45].**	Alcohol use/use disorder	Number of studies included: 27 20 human studies 7 preclinical studies Inclusion criteria: research papers, experimental and observational data discussing the association between classic psychedelic use and alcohol consumption conducted in both human and animal studies published from 2000. Exclusion criteria: review, commentary, conference and interview papers, qualitative data studies, and no full-text available articles. Studies focused on other psychedelic substances, such as MDMA or ketamine.	LSD clinical: self-reported 11 ug/kg LSD preclinical: 25–50 ug/lg Psilocybin clinical: 0.3–0.4 mg/kg Psilocybin preclinical: 1–2.5 mg/kg Ayahuasca (DMT) clinical: Ayahuasca (DMT) preclinical: 0.13–500 mg/kg No additional psychotherapies were listed for any of the interventions	LSD/Psilocybin clinical: 86.7% of participants reported a diminished effect of alcohol; 83% no longer met criteria for AUD. A total of 58% reduced or stopped alcohol consumption. LSD preclinical: alcohol consumption in mice reduced. Psilocybin clinical: mixed reports, some studies reported reduced alcohol consumption. Other studies reported no significant difference. Psilocybin preclinical: 40–50% reduction in alcohol consumption. Ayahuasca (DMT) clinical: 46.3% reduced alcohol consumption. Generally associated with improvement in AUD outcomes. One study reported 89.3% of participants consuming alcohol in past 30 days with 31.4% of participants reporting binge drinking behavior. Ayahuasca (DMT) preclinical: prevented alcohol-induced behavioral sensitization. Mescaline clinical: n individuals with previous AUD, 76% reported improvements in alcohol consumption. Another study reported increased alcohol consumption in youth.
**Juan José Fuentes et al. [50].**	Patients with a diagnosis of mental illness (neurotic symptoms, anxiety associated with life-threatening diseases) Alcohol/AUD and opioid (heroin) use disorder	Number of studies included: 11 Inclusion criteria: randomized controlled clinical trials involving patients with a diagnosis of mental illness. Exclusion criteria: experimental studies in healthy volunteers, trials with no control group or not randomized, animal studies, observational studies, review papers, qualitative studies, case reports, opinion pieces or comments, letters or editorials, conference abstract, posters and books chapters.	LSD clinical: 20–800 mcg/kg with group or individual therapy and without therapy	The vast majority of studies showed positive short-term changes in alcohol use patterns, abstinence, and reduced drinking behaviors following LSD intervention. Some studies, however, found no significant difference.
**Alessio Mosca et al. [47].**	Alcohol, opioids/heroin, cocaine, cannabis, crack, psilocybin, LSD, benzodiazepine, amphetamine, ecstasy, morphine, mushrooms	Number of studies included: 31 Included: case report/series, open-label studies, observational study, survey, double-blind placebo-controlled study. Exclusion criteria: non-original studies (e.g., review, commentary, editorial, book chapter); non-full-text articles (e.g., conference abstract); language other than English; animal/in vitro studies: articles not dealing with ibogaine/noribogaine; articles not dealing with SUD treatment	Ibogaine clinical: 70 mg/kg no additional psychotherapy listed	The results show some efficacy of ibogaine in the treatment of SUDs, but its cardiotoxicity and mortality are concerning. Some studies included showed opioid use cessation and reduced opioid withdrawal symptoms, reduced alcohol cravings and related outcomes. Many studies reported severe adverse side effects of ibogaine and noribogaine, including cardiac toxicity (cardiac arrest, bradycardia, QT prolongation, Torsade de Pointes), multi-organ failure, and death.
**Angela N. Phan and Garth E. Terry [51].**	Cannabis use disorder	Number of studies included: 4 Inclusion criteria: primary research and reports in individuals with or at risk for cannabis use disorder (e.g., individuals who use cannabis heavily or frequently, cannabis abuse, cannabis dependence), and in whom cannabis use and/or risks associated with CUD were reported before and after receipt of a psychedelic. Exclusion criteria: literature that included psychedelics and CUD that are without direct interaction (i.e., indirectly described in the clinical sample but not under study, and/or cannabis use, or risks associated with CUD were not reported after psychedelic use or administration), observational or epidemiological studies evaluating polysubstance abuse that included cannabis and repeated or chronic use of psychedelics (e.g., cohort description of cannabis use in a study of those who use MDMA regularly). Reviews and basic science (e.g., animal studies).	MDMA clinical: 40–180 mg/kg with psychotherapy LSD clinical: self-reported, no additional psychotherapy reported	MDMA clinical: No significant difference. LSD clinical: Cannabis use reduced.
**Lucas Silva Rodrigues et al. [49].**	Amphetamine and Alcohol	Number of studies included: 9 Inclusion criteria: randomized and open-label clinical studies using ayahuasca or any of its alkaloids in the treatment of SUDs; observational studies describing the use of ayahuasca or any of its alkaloids in the treatment of SUDs; quantitative studies with a statistical analysis comparing two or more groups at the same time or at least two different time points in the same group; studies that used standardized instruments to assess the pattern of use of associated substances or symptoms, preclinical studies using ayahuasca or any of its alkaloids in experimental models of SUDs, studies published between 2016 and 2020; and studies published in English, Spanish or Portuguese. Exclusion criteria: studies used as results from the previous review	Ayahuasca clinical: dose not listed and no additional psychotherapy Ayahuasca preclinical: 0.26–2 mL/kg, 30–300 mg/kg	Ayahuasca clinical: reduced alcohol and tobacco consumption, reduced prevalence of AUD, and significant decrease in dependency. Ayahuasca preclinical: prevented preference for amphetamines, reduced psychomotor agitation caused by amphetamines, decreased alcohol-induced conditioned place preference. Ayahuasca seems to prevent an increase in Fos levels in the nucleus accumbens after re-exposure to methylphenidate.
**Raman Sharma et al. [53].**	Alcohol, Opioid, Nicotine and Cocaine Problematic substance use	Number of studies included: 7 studies across 10 papers Inclusion criteria: empirical studies using any study design as long as SUD or substance misuse was the primary diagnosis of the participants in the studies; they were included regardless of the presence of other comorbid physical or mental health problems. Exclusion criteria: if there was insufficient or unclear information on treatment regimen using psychedelics (e.g., substance used, and dosage) or psychedelic-assisted psychotherapy (e.g., type of psychotherapy, duration, frequency), prohibiting reliable replication of treatment and rigorous assessment of study against eligibility criteria. Also excluded were non-primary studies, ineligible population, or intervention, no usable outcome data, ongoing studies and unable to access.	Psilocybin clinical: 0.3–70 mg/kg, with and without psychotherapy Ibogaine clinical: 3–12 mg/kg, with and without additional psychotherapy Noribogaine clinical: 60–240 mg, no additional psychotherapy Ayahuasca clinical: dose not listed but included psychotherapy	Psilocybin clinical: decreased number of alcohol drinking days and heavy drinking days. Reduced cravings and improved alcohol abstinence. Also reduced nicotine withdrawals and cravings and improved abstinence. Ibogaine clinical: improved opioid abstinence and opioid withdrawal. Noribogaine clinical: withdrawal increased prior to medications for opioid use disorder. Ayahuasca clinical: reduced alcohol, nicotine, and cocaine use. No difference in opioid use, and increased hallucinogen use. Useful for drug problems = 91.7% -Given insight into self-destructive behaviors = 86.7% -Mindful of need to become sober/abstinent now = 68.3%
**Dakota Sicignano et al. [46].**	Alcohol use disorder	Number of studies included: 6 Inclusion criteria: randomized controlled trials (RCTs) where >90% of the participants had alcohol use disorder and where a psychedelic therapy (LSD, MDMA, psilocybin, mescaline, ayahuasca, dipropyltryptamine or dimethyltrypamine) was compared to any control. Exclusion criteria: observational studies, reviews, letters to editor, and conference abstracts, written in any language other than English and trials conducted entirely in patients with a history of schizophrenia or psychosis.	Psilocybin clinical: 25/70 mg/kg–25–40/70 mg/kg, no additional psychotherapy LSD clinical: 3–800 ug, no additional psychotherapy	Psilocybin clinical: associated with abstinence or substantial reductions in alcohol use. LSD clinical: abstinence or substantial reductions in alcohol use.
**S. K. Spoelstra et al. [48].**	Nicotine and tobacco use disorder (smoking)	Number of studies included: 8 Inclusion criteria: research that focused on the relation between the use of classic or non-classic psychedelics and outcomes related to nicotine smoking (no other addictions), utilized qualitative or quantitative empirical data (both clinical and non-clinical studies were included, was published in English and peer reviewed Exclusion criteria: case reports, systematic reviews, and case studies, or studies with no focus on smoking	Ayahuasca clinical: no dose and no additional psychotherapy Psilocybin clinical: some with and some without psychotherapy, most doses were not listed and only one study used a precise dose of 20–30/70 mg/kg Psychedelics clinical: (“magic”) mushrooms, LSD, morning glory seeds, mescaline, peyote cactus, San Pedro cactus, dimethyltryptamine (DMT), and Ayahuasca	Ayahuasca clinical: 69.2% of participants self-reported quitting smoking, 18.3% reported reducing smoking, and 12.5% reported quitting for a period of time, then returning to tobacco smoking. Psilocybin clinical: 67% of participants were abstainers; another study reported 80% abstinence at 6-month follow-up. Psychedelics clinical: 38% of participants reported continuous smoking cessation.
**Alexander Trope et al. [54].**	Alcohol use disorder	Number of studies included: 12 Inclusion criteria: article that contained some description of group methods, demographic and diagnostic information, and quantified outcome data. Exclusion criteria: inability to locate the abstract or full text in English or Spanish	LSD clinical: 25–1000 ug, with psychotherapy	LSD clinical: no significant differences in abstinence of alcohol between groups over 6–18-month follow-up. Cannot reliably determine the safety of interventions given that the assessment and reporting of adverse events were inconsistent.
**Pim B. van der Meer et al. [52].**	Alcohol use disorder and tobacco use disorder	Number of studies included: 6 Inclusion criteria: intervention with ≥1 dose of psilocybin; clinical trial (open-label [pilot] studies, single-blind, or double-blind [placebo-controlled] trials); diagnosis of a SUD or non-substance-related disorder [i.e., diagnosed by the general practitioner or by a structured clinical interview based on Diagnostic and Statistical Manual of Mental Disorders (DSM) or International Classification of Diseases (ICD) criteria], adult patients (18 years); patients, and language of the manuscript was English, Spanish, Portuguese, Dutch, German, or French. Exclusion criteria: animal studies; experimental studies in healthy volunteers; observational studies; review papers; qualitative studies; opinion pieces or comments; letters or editorials; conference abstracts or posters; and case reports.	Psilocybin clinical: 6–70 mg with and without psychotherapy LSD clinical: 100–800 mcg	Psilocybin clinical: all four clinical trials indicated a beneficial effect of psilocybin-assisted therapy on SUD symptoms. Number of heavy alcohol drinking days reduced, and abstinence increased. Nicotine abstinence increased—7-day point prevalence abstinence 80% at week 26 and 67% at week 52 based on both biomarkers and self-report. Both studies that alternated LSD and psilocybin as well as included or did not include psychotherapy resulted in improvements in alcohol and nicotine use disorder.

Abbreviations: AUD = alcohol use disorder; CUD = cannabis use disorder; LSD—lysergic acid diethylamide; DMT—N, N-dimethyltryptamine; MDMA—3,4-methylenedioxy-methylamphetamine.

**Table 3:** Ketamine.

Authors	Target Condition	Inclusion/Exclusion Criteria	Interventions	Outcomes
**Kelson et al. (2023) [55].**	AUD	Number of studies included: 11 studies with 854 participants Inclusion: adults diagnosed with AUD; studies assessing ketamine efficacy for AUD. Exclusion: non-AUD populations: studies focused solely on ketamine metabolites.	Ketamine IV: 0.5–2.0 mg/kg. Psychotherapy, behavioral therapy, standard AUD medications (acamprosate, naltrexone, disulfiram).	The results were mixed with respect to relapse, craving, and withdrawal symptoms. Ketamine may improve heavy drinking and increase the number of post-infusion abstinent days. It may be an effective therapeutic modality for people with alcohol use disorders who fail to respond to FDA-approved first-line agents.
**Garel et al. (2022) [57]**	AUD	Number of studies included: 8 studies, 634 participants Inclusion: studies examining ketamine use for AUD; short-term and long-term alcohol consumption reduction. Exclusion: non-peer-reviewed studies; non-human studies.	Ketamine IV: 0.5–2.0 mg/kg. Psychotherapy, psychedelics, alcohol-related memory retrieval exercises.	Ketamine interventions appear to be safe and possibly effective for alcohol use/abstinence and cravings. Equivocal evidence related to alcohol withdrawal management. All the studies were determined to be at critical risk of bias.
**Jones et al. (2018) [41]**	CUD, OUD, cannabis use disorder	Number of studies included: 7 studies—2 on AUD, 2 on cocaine use disorder, and 3 on OUD Inclusion: studies on ketamine for stimulant and OUD; impact on cravings and abstinence. Exclusion: studies lacking ketamine treatment; non-human trials.	Ketamine IV: 0.4–2.0 mg/kg. Ketamine IM: 2–3 mg/kg. Behavioral therapy, psychotherapy, pharmacotherapy for OUD, psychedelic-assisted therapy.	Both cocaine studies found improvements in cravings and decreased cocaine use rates. Studies of alcohol and opioid use disorders found improvement in abstinence rates in the ketamine group, with significant between-group effects noted for up to two years following a single infusion. Results suggest that ketamine may facilitate abstinence across multiple substances of abuse.
**Kew et al. (2023) [58]**	PTSD, anxiety, OCD	Number of studies included: 19 studies (*n* = 1008 participants)—7 studies examined ketamine for the treatment of SUD—*n* = 3 for AUD, *n* = 2 for OUD (“heroin dependence”), *n* = 1 for cocaine use disorder, *n* = 1 for cannabis use disorder Inclusion: studies combining ketamine with psychotherapy for psychiatric disorders. Exclusion: case reports, non-human studies.	Ketamine IV: 0.5 mg/kg (includes repeated infusions). CBT, supportive psychotherapy, group therapy.	For AUD, ketamine treatment performed better than saline control and treatment as usual. For OUD (heroin dependence), patients allocated to higher-dose ketamine and repeated ketamine/psychotherapy groups had better outcomes (abstinence). Abstinence from cocaine was observed in 48.2% of patients receiving ketamine and MBRP compared with 10.7% of patients receiving midazolam and MBRP. Twelve days post-infusion, there was a significantly greater proportion of cannabis-abstinent patients in the ketamine group compared with the midazolam group.
**Walsh et al. (2022) [59]**	Mental health, SUDs	Number of studies included: 83 published reports—33 systematic reviews, 29 RCT, two randomized trials without placebo, three non-randomized trials with controls, six open-label trials and 10 retrospective reviews. 14 studies examining ketamine as a treatment for SUD, including six studies focusing on AUD, five on cocaine use disorder, and three on OUD Inclusion: human studies on ketamine for mental health disorders, systematic reviews, meta-analyses. Exclusion: animal studies.	Ketamine IV, IM, and IN: 0.4–3 mg/kg. Psychotherapy, CBT, standard mental health pharmacotherapies.	Systematic reviews and meta-analyses provide support for robust, rapid and transient antidepressant and anti-suicidal effects of ketamine. Evidence for other indications is less robust, although it suggests similarly positive yet short-lived effects.
**Drozdz et al. (2022) [56]**	KAP for SUD and mental health disorders	Number of studies included: 17 (7 RCT, 5 case studies or case series; 4 were open-label trials; and 1 retrospective study.) Inclusion: studies reporting on ketamine-assisted psychotherapy for pain, mental health, and SUDs. Exclusion: animal studies; studies focused on recreational ketamine use.	ketamine IV, IM, IN, and SL: 0.5–2.0 mg/kg (doses varied). Psychotherapy, psychedelic-assisted therapy, opioid tapering programs.	Ketamine in combination with psychotherapy appears to promote abstinence, enhance motivation, and improve cravings and relapse prevention in AUD, cocaine use disorder, cannabis use disorder, and OUD.

Abbreviations: AUD = alcohol use disorder; IV = intravenous; CUD = cocaine use disorder; OUD = opioid use disorder; IM = intramuscular; PTSD = post-traumatic stress disorder; OCD = obsessive–compulsive disorder; CBT = cognitive behavioral therapy; SUDs = substance use disorders; IN = intranasal; KAP = ketamine assisted psychotherapy; SL = sublingual; RCT = randomized clinical trial; FDA = food and drug administration; MBRP = mindfulness-based lapse prevention.

## Data Availability

The data presented in this study are derived from previously published articles and are publicly available in the respective publications cited throughout the manuscript.

## Supplementary Materials

The following supporting information can be downloaded at https://www.mdpi.com/article/10.3390/brainsci15101056/s1, Search Strategy.
