# Supplementary material for: The Role of Psychedelics in the Treatment of Substance Use Disorders: An Overview of Systematic Reviews
Source: Brain Sci. 2025 Sep 28;15(10):1056. doi: 10.3390/brainsci15101056 (PMC12562643; doi:10.3390/brainsci15101056)
Supplement: Supplementary file 1 [file brainsci-15-01056-s001.zip › brainsci-3790534-supplementary.docx]

**SUPPLEMENTARY MATERIALS**

**The Role of Psychedelics in the Treatment of Substance Use Disorders: an overview of systematic reviews.**

**SEARCH STRATEGY**

**Development**

The search strategy was developed in Embase (Ovid) by a medical librarian (MM).

**Limits and filters**

English language database limits were applied as available or built into searches when possible. An unpublished filter was used to limit study design to systematic review with or without meta-analysis. Conference abstracts and conference reviews were excluded via command line search. Subject areas and categories were utilized as limits in Scopus and Web of Science databases.

**Peer Review**

The Embase search query was reviewed by an independent medical librarian.

*DATABASES, SEARCH ENGINES, AND TRIAL REGISTER SEARCHES*

**Embase 1974 to 2024 December 19 (Wolters Kluwer Ovid interface)**

Date searched: December 20, 2024

Records retrieved: 347

Language: limited to English

Study design: limited to systematic review with or without meta-analysis

Publication type: excluded conference abstract and conference review

**((**(alcoholism/ OR amphetamine dependence/ OR benzodiazepine dependence/ OR cocaine dependence/ OR drug dependence/ OR methamphetamine dependence/ OR exp narcotic dependence/ OR phencyclidine dependence/ OR alcoholism.ti,ab,kf. OR (addict OR addicts OR alcoholic*).ti,kf OR ((alcohol or amphetamine* OR anxiolytic* OR barbiturate* OR benzo* OR cocaine OR ethanol OR fentanyl OR (glue adj1 sniff*) OR heroin or huffing* OR hypnotic* OR inhalant* OR meth OR methamphetamine* OR morphine OR narcotic* OR opioid* OR opiate* OR opium OR PCP OR phencyclidine OR sedative* OR stimulant*) adj5 (addict* OR dependen*)).ti,ab. OR ((drug* OR substance*) ADJ3 (addict* OR dependen*)).ti,ab,kf. OR ((alcohol or amphetamine* OR anxiolytic* OR barbiturate* OR benzo* OR cocaine OR ethanol OR fentanyl OR (glue adj1 sniff*) OR heroin or huffing* OR hypnotic* OR inhalant* OR meth OR methamphetamine* OR morphine OR narcotic* OR opioid* OR opiate* OR opium OR PCP OR phencyclidine OR sedative* OR stimulant*) AND (dependen*)).kf. OR use-disorder*.ti,ab,hw,kf,dq.)**)** **AND (**(cannabinoid/dt OR cannabis/dt OR ketamine/dt OR medical cannabis/dt OR nitrous oxide/dt OR exp psychedelic agent/dt OR psychedelic therapy/ OR (((medical OR medicin*) ADJ2 (cannabis OR mari#uana)) OR ((cannabinoid* OR cannabis OR DMT OR dimethyltryptamine OR hallucinogen* OR ketamine OR LSD OR lysergic-acid-diethylamide OR mari#uana OR MDMA OR methylenedioxymethamphetamine OR nitrous-oxide OR psilocybin* OR psych#delic*) ADJ3 (pharmacotherap* OR therap* OR treat*))).ti,ab,kf,dq.) **AND (**((Systematic OR metaanalysis OR meta-analysis) ADJ (review)).ti,ab OR systematic review.pt OR meta analysis.pt OR (Cochrane OR Embase OR MEDLINE OR PubMed OR Scopus OR PRISMA).ab.**) AND (**English.la**) NOT** (conference abstract OR conference review).pt.

**Ovid MEDLINE(R) 1946 to Present and Epub Ahead of Print, In-Process & Other Non-Indexed Citations and Ovid MEDLINE(R) Daily**

Date searched: December 20, 2024

Records retrieved: 247

Language: limited to English

Study design: limited to systematic review with OR without meta-analysis

**((**(alcoholism/ OR amphetamine-related disorders/ OR cocaine-related disorders/ OR narcotic-related disorders/ OR opioid-related disorders/ OR substance-related disorders/ OR alcoholism.ti,ab,kf. OR (addict OR addicts OR alcoholic*).ti,kf OR ((alcohol or amphetamine* OR anxiolytic* OR barbiturate* OR benzo* OR cocaine OR ethanol OR fentanyl OR (glue adj1 sniff*) OR heroin or huffing* OR hypnotic* OR inhalant* OR meth OR methamphetamine* OR morphine OR narcotic* OR opioid* OR opiate* OR opium OR PCP OR phencyclidine OR sedative* OR stimulant*) adj5 (addict* OR dependen*)).ti,ab. OR ((drug* OR substance*) ADJ3 (addict* OR dependen*)).ti,ab,kf. OR ((alcohol or amphetamine* OR anxiolytic* OR barbiturate* OR benzo* OR cocaine OR ethanol OR fentanyl OR (glue adj1 sniff*) OR heroin or huffing* OR hypnotic* OR inhalant* OR meth OR methamphetamine* OR morphine OR narcotic* OR opioid* OR opiate* OR opium OR PCP OR phencyclidine OR sedative* OR stimulant*) AND (dependen*)).kf. OR use-disorder*.ti,ab,hw,kf.)**)** **AND** **(**(cannabinoids/tu OR cannabis/tu OR exp hallucinogens/tu OR ketamine/tu OR medical marijuana/tu OR nitrous oxide/tu OR (((medical OR medicin*) ADJ2 (cannabis OR mari#uana)) OR ((cannabinoid* OR cannabis OR DMT OR dimethyltryptamine OR hallucinogen* OR ketamine OR LSD OR lysergic-acid-diethylamide OR mari#uana OR MDMA OR methylenedioxymethamphetamine OR nitrous-oxide OR psilocybin* OR psych#delic*) ADJ3 (pharmacotherap* OR therap* OR treat*))).ti,ab,kf.) **AND** **(**((Systematic OR metaanalysis OR meta-analysis) ADJ (review)).ti,ab OR systematic review.pt OR meta analysis.pt OR (Cochrane OR Embase OR MEDLINE OR PubMed OR Scopus OR PRISMA).ab.**) AND** (English.la)

**APA PsycINFO 1806 to December 2024 Week 3 (Wolters Kluwer Ovid interface)**

Date searched: December 20, 2024

Records retrieved: 104

Language: limited to English

Study design: limited to systematic review with or without meta-analysis

**((**(alcoholism/ OR “alcohol use disorder”/ OR drug addiction/ OR drug dependency/ OR exp “opioid use disorder”/ OR “substance use disorder”/ OR alcoholism.ti,ab,id. OR (addict OR addicts OR alcoholic*).ti,id OR ((alcohol or amphetamine* OR anxiolytic* OR barbiturate* OR benzo* OR cocaine OR ethanol OR fentanyl OR (glue adj1 sniff*) OR heroin or huffing* OR hypnotic* OR inhalant* OR meth OR methamphetamine* OR morphine OR narcotic* OR opioid* OR opiate* OR opium OR PCP OR phencyclidine OR sedative* OR stimulant*) adj5 (addict* OR dependen*)).ti,ab. OR ((drug* OR substance*) ADJ3 (addict* OR dependen*)).ti,ab,id. OR ((alcohol or amphetamine* OR anxiolytic* OR barbiturate* OR benzo* OR cocaine OR ethanol OR fentanyl OR (glue adj1 sniff*) OR heroin or huffing* OR hypnotic* OR inhalant* OR meth OR methamphetamine* OR morphine OR narcotic* OR opioid* OR opiate* OR opium OR PCP OR phencyclidine OR sedative* OR stimulant*) AND (dependen*)).id. OR use-disorder*.ti,ab,hw,id.)**)** **AND** **(**(medical marijuana/ OR psychedelic assisted therapy/ OR (((medical OR medicin*) ADJ2 (cannabis OR mari#uana)) OR ((cannabinoid* OR cannabis OR DMT OR dimethyltryptamine OR hallucinogen* OR ketamine OR LSD OR lysergic-acid-diethylamide OR mari#uana OR MDMA OR methylenedioxymethamphetamine OR nitrous-oxide OR psilocybin* OR psych#delic*) ADJ3 (pharmacotherap* OR therap* OR treat*))).ti,ab,id.) **AND (**((Systematic OR metaanalysis OR meta-analysis) ADJ (review)).ti,ab OR systematic review.pt OR meta analysis.pt OR (Cochrane OR Embase OR MEDLINE OR PubMed OR Scopus OR PRISMA).ab.**) AND** (English.la)

**EBM Reviews - Cochrane Database of Systematic Reviews 2005 to December 18, 2024 (Wolters Kluwer Ovid interface)**

Date searched: December 20, 2024

Records retrieved: 5

**((**alcoholism.ti,ab,kw. OR (addict OR addicts OR alcoholic*).ti,kw OR ((alcohol or amphetamine* OR anxiolytic* OR barbiturate* OR benzo* OR cocaine OR ethanol OR fentanyl OR (glue adj1 sniff*) OR heroin or huffing* OR hypnotic* OR inhalant* OR meth OR methamphetamine* OR morphine OR narcotic* OR opioid* OR opiate* OR opium OR PCP OR phencyclidine OR sedative* OR stimulant*) adj5 (addict* OR dependen*)).ti,ab. OR ((drug* OR substance*) ADJ3 (addict* OR dependen*)).ti,ab,kw. OR ((alcohol or amphetamine* OR anxiolytic* OR barbiturate* OR benzo* OR cocaine OR ethanol OR fentanyl OR (glue adj1 sniff*) OR heroin or huffing* OR hypnotic* OR inhalant* OR meth OR methamphetamine* OR morphine OR narcotic* OR opioid* OR opiate* OR opium OR PCP OR phencyclidine OR sedative* OR stimulant*) AND (dependen*)).kw. OR use-disorder*.ti,ab,kw.**)** **AND** **(**(((medical OR medicin*) ADJ2 (cannabis OR mari#uana)) OR ((cannabinoid* OR cannabis OR DMT OR dimethyltryptamine OR hallucinogen* OR ketamine OR LSD OR lysergic-acid-diethylamide OR mari#uana OR MDMA OR methylenedioxymethamphetamine OR nitrous-oxide OR psilocybin* OR psych#delic*) ADJ3 (pharmacotherap* OR therap* OR treat*))).ti,ab,kw.

**Scopus**

Date searched: December 20, 2024

Records retrieved: 296

Language: limited to English

Subject Areas: limited to Medicine; Neuroscience; Pharmacology, Toxicology and Pharmaceutics; and Psychology

**((**TITLE-ABS-KEY (alcoholism) OR TITLE (addict OR addicts OR alcoholic) OR KEY (addict OR addicts OR alcoholic) OR TITLE-ABS ((alcohol or amphetamine OR anxiolytic OR barbiturate OR benzo OR cocaine OR ethanol OR fentanyl OR (glue W/1 sniff*) OR heroin or huffing OR hypnotic OR inhalant OR meth OR methamphetamine OR morphine OR narcotic OR opioid OR opiate OR opium OR PCP OR phencyclidine OR sedative OR stimulant) W/4 (addict* OR dependen*)) OR TITLE-ABS-KEY ((drug* OR substance*) W/2 (addict* OR dependen*)) OR KEY ((alcohol or amphetamine OR anxiolytic OR barbiturate OR benzo OR cocaine OR ethanol OR fentanyl OR (glue W/1 sniff*) OR heroin or huffing OR hypnotic OR inhalant OR meth OR methamphetamine OR morphine OR narcotic OR opioid OR opiate OR opium OR PCP OR phencyclidine OR sedative OR stimulant) AND (dependen*)) OR TITLE-ABS-KEY (use-disorder)**) AND (**TITLE-ABS-KEY (((medical OR medicin*) W/1 (cannabis OR marijuana)) OR ((cannabinoid OR cannabis OR DMT OR dimethyltryptamine OR hallucinogen* OR ketamine OR LSD OR lysergic-acid-diethylamide OR marijuana OR MDMA OR methylenedioxymethamphetamine OR nitrous-oxide OR psilocybin OR psychedelic) W/3 (pharmacotherap* OR therap* OR treat*))) **AND** (TITLE-ABS ((Systematic OR metaanalysis OR meta-analysis) PRE/0 (review)) OR ABS (Cochrane OR Embase OR MEDLINE OR PubMed OR Scopus OR PRISMA)) AND (LIMIT-TO (LANGUAGE , "English")) AND (LIMIT-TO (LANGUAGE, "English")) AND (LIMIT-TO (SUBJAREA, "MEDI") OR LIMIT-TO (SUBJAREA, "NEUR") OR LIMIT-TO (SUBJAREA, "PHAR") OR LIMIT-TO (SUBJAREA, "PSYC"))

**Science Citation Index Expanded (SCI-Expanded)—1975-present (Web of Science)**

Date searched: December 20, 2024

Records retrieved: 193

Language: limited to English

Categories: limited to Behavioral Sciences; Clinical Neurology; Medicine General Internal; Neurosciences; Pharmacology Pharmacy; Psychiatry; Psychology; Psychology Clinical; Substance Abuse.

Document Types: excluded meeting abstracts

**(((**TS=(alcoholism) OR TI=(addict OR addicts OR alcoholic) OR AK=(addict OR addicts OR alcoholic) OR TI=((alcohol or amphetamine OR anxiolytic OR barbiturate OR benzo OR cocaine OR ethanol OR fentanyl OR (glue NEAR/1 sniff*) OR heroin or huffing OR hypnotic OR inhalant OR meth OR methamphetamine OR morphine OR narcotic OR opioid OR opiate OR opium OR PCP OR phencyclidine OR sedative OR stimulant) NEAR/4 (addict* OR dependen*)) OR AB=((alcohol or amphetamine OR anxiolytic OR barbiturate OR benzo OR cocaine OR ethanol OR fentanyl OR (glue NEAR/1 sniff*) OR heroin or huffing OR hypnotic OR inhalant OR meth OR methamphetamine OR morphine OR narcotic OR opioid OR opiate OR opium OR PCP OR phencyclidine OR sedative OR stimulant) NEAR/4 (addict* OR dependen*)) OR TS=((drug* OR substance*) NEAR/2 (addict* OR dependen*)) OR AK=((alcohol or amphetamine OR anxiolytic OR barbiturate OR benzo OR cocaine OR ethanol OR fentanyl OR (glue W/0 sniff*) OR heroin or huffing OR hypnotic OR inhalant OR meth OR methamphetamine OR morphine OR narcotic OR opioid OR opiate OR opium OR PCP OR phencyclidine OR sedative OR stimulant) AND (dependen*)) OR TS=(use-disorder)**) AND (**TS=(((medical OR medicin*) NEAR/1 (cannabis OR marijuana)) OR ((cannabinoid OR cannabis OR DMT OR dimethyltryptamine OR hallucinogen* OR ketamine OR LSD OR lysergic-acid-diethylamide OR marijuana OR MDMA OR methylenedioxymethamphetamine OR nitrous-oxide OR psilocybin OR psychedelic) NEAR/3 (pharmacotherap* OR therap* OR treat*))) **AND (**TI=((Systematic OR metaanalysis OR meta-analysis) NEAR/0 (review)) OR AB=(Cochrane OR Embase OR MEDLINE OR PubMed OR Scopus OR PRISMA OR ((Systematic OR metaanalysis OR meta-analysis) NEAR/0 (review)))**)**

**Emerging Sources Citation Index (ESCI)—2018-present (Web of Science)**

Date searched: December 20, 2024

Records retrieved: 17

Language: limited to English

Categories: limited to Clinical Neurology; Medicine General Internal; Neurosciences; Pharmacology Pharmacy; Psychiatry; Psychology Clinical; and Substance Abuse.

**(((**TS=(alcoholism) OR TI=(addict OR addicts OR alcoholic) OR AK=(addict OR addicts OR alcoholic) OR TI=((alcohol or amphetamine OR anxiolytic OR barbiturate OR benzo OR cocaine OR ethanol OR fentanyl OR (glue NEAR/1 sniff*) OR heroin or huffing OR hypnotic OR inhalant OR meth OR methamphetamine OR morphine OR narcotic OR opioid OR opiate OR opium OR PCP OR phencyclidine OR sedative OR stimulant) NEAR/4 (addict* OR dependen*)) OR AB=((alcohol or amphetamine OR anxiolytic OR barbiturate OR benzo OR cocaine OR ethanol OR fentanyl OR (glue NEAR/1 sniff*) OR heroin or huffing OR hypnotic OR inhalant OR meth OR methamphetamine OR morphine OR narcotic OR opioid OR opiate OR opium OR PCP OR phencyclidine OR sedative OR stimulant) NEAR/4 (addict* OR dependen*)) OR TS=((drug* OR substance*) NEAR/2 (addict* OR dependen*)) OR AK=((alcohol or amphetamine OR anxiolytic OR barbiturate OR benzo OR cocaine OR ethanol OR fentanyl OR (glue W/0 sniff*) OR heroin or huffing OR hypnotic OR inhalant OR meth OR methamphetamine OR morphine OR narcotic OR opioid OR opiate OR opium OR PCP OR phencyclidine OR sedative OR stimulant) AND (dependen*)) OR TS=(use-disorder)**) AND (**TS=(((medical OR medicin*) NEAR/1 (cannabis OR marijuana)) OR ((cannabinoid OR cannabis OR DMT OR dimethyltryptamine OR hallucinogen* OR ketamine OR LSD OR lysergic-acid-diethylamide OR marijuana OR MDMA OR methylenedioxymethamphetamine OR nitrous-oxide OR psilocybin OR psychedelic) NEAR/3 (pharmacotherap* OR therap* OR treat*))) **AND (**TI=((Systematic OR metaanalysis OR meta-analysis) NEAR/0 (review)) OR AB=(Cochrane OR Embase OR MEDLINE OR PubMed OR Scopus OR PRISMA OR ((Systematic OR metaanalysis OR meta-analysis) NEAR/0 (review)))**)**
